# The Development of Text Messages to Support People at Risk of Diabetes in Low-Resourced Communities: The South African Diabetes Prevention Programme

**DOI:** 10.3390/nu15214692

**Published:** 2023-11-05

**Authors:** Jillian Hill, Mieke Faber, Cindy George, Nasheeta Peer, Tshavhuyo Mulabisano, Sonja Mostert, Eugene Sobngwi, Andre P. Kengne

**Affiliations:** 1 Non-Communicable Diseases Research Unit, South African Medical Research Council (SAMRC), Cape Town 7505, South Africa; info@mrc.ac.za (M.F.); cindy.george@mrc.ac.za (C.G.); nasheeta.peer@mrc.ac.za (N.P.); tshavhuyo.mulabisano@mrc.ac.za (T.M.); sonja.mostert@samrc.ac.za (S.M.); andre.kengne@mrc.ac.za (A.P.K.); 2 Center of Excellence for Nutrition, North-West University, Potchefstroom 2531, South Africa; 3 Department of Medicine, Faculty of Health Sciences, University of Cape Town, Cape Town 7700, South Africa; 4 Department of Medicine, University of Yaounde, Yaounde 00237, Cameroon; sobngwieugene@yahoo.fr

**Keywords:** text message development, diabetes prevention, lifestyle behavior

## Abstract

Emerging evidence suggests that the addition of text messages to standard healthy lifestyle interventions may improve the outcomes of diabetes prevention programs (DPP). This paper describes the process of developing text messages targeting behavior change in people at risk of developing diabetes in low-resourced communities as part of the South African DPP (SA-DPP). The development comprised multiple steps led by nutrition and physical activity experts. The steps included the following: (1) text message development based on the existing SA-DPP curriculum and its formative research; (2) text message evaluation for readability/understandability in terms of content, language, and quality, with 75 participants from two low-resourced areas in Cape Town; (3) text message refinement by the expert panel; (4) evaluation of the refined text messages by participants from Step 2; and (5) text bank finalization. Based on the readability survey, 37 of the 67 formulated text messages [24 of the 44 encouraged healthy eating, and 13 of the 23 promoted physical activity] were refined. Based on focused discussions with participants, seven more messages were refined to consider alternative terminology. The final text bank includes a total of 67 messages comprising topics related to fruit and vegetable consumption as well as the importance of having variety in the diet (n = 15), limiting fat intake (n = 10), avoiding sugar (n = 11), avoiding salt (n = 5), promoting fiber-rich foods (n = 1), messages promoting physical activity (n = 21), and general check-in messages (n = 4). Most of the text messages were acceptable, understandable, and largely feasible to all participants, with some of the nutrition-related messages being less feasible for participants due to their socioeconomic position. The next step is to assess the text messages in the SA-DPP intervention trial.

## 1. Introduction

Type 2 diabetes is a leading contributor to noncommunicable disease (NCD)-related mortality globally [1]. According to the 10th Edition of the International Diabetes Federation’s Diabetes Atlas, 537 million people aged between 20 and 79 years were living with type 2 diabetes in 2021, with 24 million of these individuals residing in sub-Saharan Africa (SSA) [2]. South Africa has the largest population of people living with type 2 diabetes in SSA with approximately 4.2 million people between the ages of 20 and 79 years having diabetes [2]. Diabetes is currently the sixth leading cause of death in South Africa [3] and accounts for 7% of NCD-related mortality [4].

Diabetes prevention programs have proven to be successful to varying degrees, with the most successful being the Diabetes Prevention Program (DPP), a randomized clinical trial in the United States, which has reduced diabetes incidence by 58% [5,6,7]. However, a major challenge exists in translating individual-level diabetes prevention strategies to large sub-groups, i.e., the successful lifestyle modification interventions employed in clinical trials are labor-intensive and expensive and, thus, difficult to operationalize at scale [8].

These challenges may be partially addressed with the use of technology such as text messaging systems. Text messaging programs related to health behaviors have been used in a variety of contexts with remarkable success, including in HIV prevention, medication adherence, pregnancy education, substance use/smoking cessation, weight loss, diabetes management, and depression [9,10]. Emerging evidence suggests that the addition of text messages to standard healthy lifestyle interventions may improve the outcomes of DPPs [9,10,11]. The high and widespread penetration of mobile phones worldwide, irrespective of socioeconomic status, enables the inclusion of text messages in DPPs. Text messages can be delivered to the most basic phones and are read within minutes of being sent. They are a useful tool to provide short, timed bursts of information throughout the day, constantly reiterating healthy behavior goals [12,13].

The South African DPP (SA-DPP) intervention strategy attempts to overcome the issue of scalability by employing a low-intensity group-based lifestyle modification program supported by the delivery of a short messaging service (SMS) to prevent diabetes in South Africa. Although text messaging to encourage behavior change is becoming more popular, Willoubhy et al. (2015) [14], Abroms et al. (2015) [15], and Para et al. (2018) [16] have noted that the development and pretesting of these text messages have been unclear. These authors highlight the underreporting and underdevelopment of these text messages, i.e., the scarcity of publications documenting the process as well as the lack of rigor in the development process of those published [14]. Also, research investigating text messaging in low-income countries is specifically scarce [13]. This study was thus informed by recommendations from authors who recognized the gap in the reporting of the development and pretesting of text messages for health behavior change [13,14,15,16]. The authors’ recommendations included conducting formative research, message development, pretesting, and validation as well as piloting. The purpose of the text messages in the SA-DPP intervention is to serve as support during its maintenance phase (as part of the transtheoretical model’s stages of change [17]) after the six face-to-face sessions.

## 2. Methods and Processes

### 2.1. Brief Description of the South African Diabetes Prevention Program Intervention

The SA-DPP is a lifestyle intervention targeting individuals at high risk of developing type 2 diabetes mellitus. The SA-DPP lifestyle intervention includes six group sessions based on empowerment ideology, emphasizing the participant’s ability to make informed decisions and his/her role as an independent decision maker who takes responsibility and regulates his/her own actions [18,19]. The intervention will be led by nonprofessional or community health workers (CHWs), assisted by nurses and/or dieticians as required, and will be complemented by structured mobile phone messages to augment adherence and retention. The lifestyle change objectives (diet and physical activity) of the SA-DPP are based on the original Finnish Diabetes Prevention Study, i.e., (1) <30% of total energy intake from fat; (2) <10% of total energy intake from saturated fat; (3) >15 g of fiber/1000 kcal; (4) >4 h/week moderate level of physical activity; and (5) >5% reduction in body mass index [20]. The SA-DPP was approved by the ethics committee of the South African Medical Research Council (approval No. EC018-7/2015) and conducted in accordance with the Declaration of Helsinki. Written informed consent was obtained from all participants involved in the study.

### 2.2. South African Diabetes Prevention Program Curriculum Development

A qualitative mixed-method staged approach was followed in developing the SA-DPP intervention curriculum and tools (facilitator and participant workbooks). It included 11 steps, as follows: (1) reviewing and learning from existing evidence; (2) a needs assessment; (3) expert input; (4) development of the curriculum and tools; (5) expert content evaluation; (6) design and layout of the curriculum and tools; (7) participant readability and acceptability evaluation; (8) refining the design and layout of the curriculum and tools; (9) translating the curriculum and participant workbook; (10) suitability evaluation (pilot intervention); and (11) finalizing the curriculum [21].

During the preparation phase, existing evidence on similar DPP interventions was reviewed [such as but not limited to the group-organized YMCA DPP (GO-YDPP) [22] and the Kerala Diabetes Prevention Program (K-DPP) [23], focus group discussions with individuals from the target population were conducted as part of a needs assessment, and experts were consulted. The curriculum booklet, a participant workbook, and a facilitator workbook were developed, and the content was evaluated by experts in the field. The design and layout of the booklet and workbooks needed to be culturally and contextually appropriate. The printed material was evaluated for readability and acceptability by participants from the target population. Based on their feedback, the design and layout were refined, and the printed material was translated. The suitability of the intervention was tested in a pilot study; based on feedback from the participants and facilitator, the curriculum was revised where needed and finalized. Through this process, a context-specific intervention and printed materials were developed [21]. A complete evaluation of this culturally relevant model for diabetes prevention in South Africa is pending.

### 2.3. Text Messaging Development Protocol

**Figure 1:**
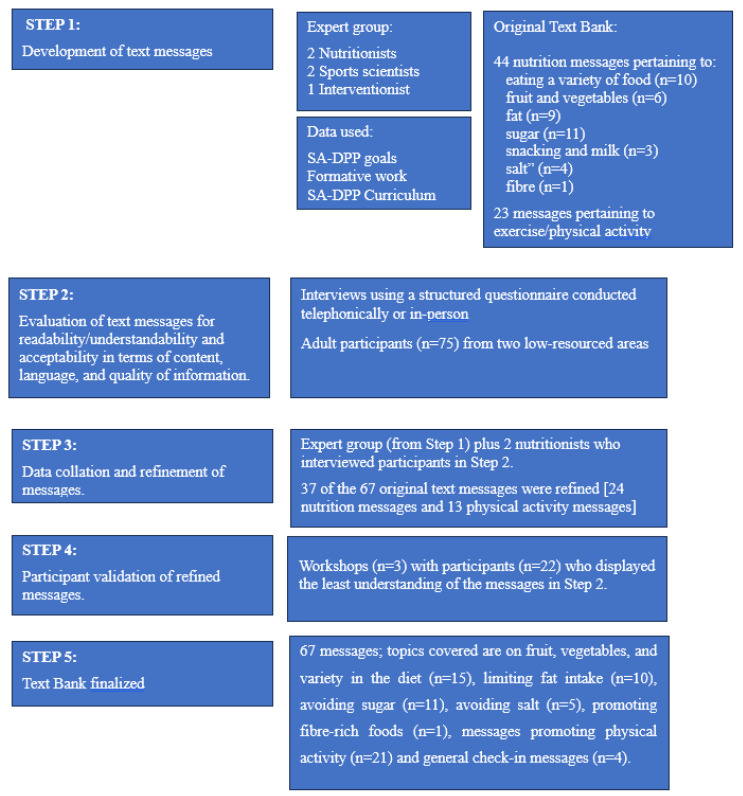
Text-messaging development process.

This arm comprised the five steps outlined below (refer to Figure 1).

#### 2.3.1. **Step 1:** Text Message Development

Two nutrition experts, two physical activity specialists, and the project manager developed 44 nutrition-related messages and 23 exercise-related messages based on the existing SA-DPP curriculum and its formative research [21]. The SA-DPP curriculum was largely informed by the South African Food-Based Dietary Guidelines (SAFBDG) [24], which are 11 brief, encouraging dietary guidelines. The goal was to empower the South African consumer to choose food and drink (based on existing eating patterns) that would contribute to a nutritionally adequate diet and lower risk for NCDs [24]. The 44 nutrition-related messages were themed as follows: 10 messages that spoke to “eating a variety of food”; six pertaining to “fruit and vegetables”; nine pertaining to “fat”; 11 pertaining to “sugar”; 3 generic messages around snacking and milk; 4 messages pertaining to “salt”; 1 message pertaining to “fiber”, and 23 messages pertaining to “exercise/physical activity”.

#### 2.3.2. **Step 2:** Text Message Evaluation

The next step included the evaluation of these text messages for readability/understandability and acceptability in terms of content, language, and quality of information. A validated tool for assessing printed material [25], previously adapted for our setting during our curriculum development process, was used. The responses to the respective questions were based on a 3-point Likert scale with options including “agree”, “partly agree”, or “disagree”. Participants’ comments on any content not well understood were also captured. Interviews using a structured questionnaire were conducted telephonically or in person with 75 participants from two low-resourced residential areas (one mixed-ancestry and one Black African group). These participants fit the same criteria as the target population of the SA-DPP and previously participated in the validation of the quantified food frequency questionnaire used in the SA-DPP study. The current validation study targeted a total of 100 respondents from both residential areas. These were divided equally by residential area with 5 groups of 10 participants targeted from each area. Only a total of 75 responded.

Each participant received 13 messages to be evaluated. These comprised 12 messages from the main text bank and a general reminder text message for eight groups (four per area). The last two groups received 13 messages from the main text bank only. The project driver delivered a survey pack consisting of an information sheet, the 13 text messages, and the evaluation form (for reference while being interviewed) to all participants.

The participants were interviewed individually by two nutritionists. Due to COVID-19 restrictions, all interviews were planned to be conducted telephonically; however, due to difficulties in getting hold of various participants, a decision was made to conduct in-person interviews, following strict COVID-19 safety protocols. Thus, the initial 40 interviews were conducted telephonically with the remaining 35 conducted in person.

*Interview process:* During the interview process, the interviewer explained the study to the participant in detail and obtained verbal and written consent for telephonic and in-person interviews, respectively. The interviewer, while administering the interview, captured the responses electronically on an Excel spreadsheet. Participants received an R50 ($3) store voucher for participation.

#### 2.3.3. **Steps 3–5**: Message Refinement

The participants’ responses to the readability/understandability and acceptability in terms of the content, language, and quality of the text message information were tallied. Messages that were not well understood were refined by the expert panel, which included the two nutritionists who conducted the interviews. Based on the comments recorded during the interviews, the interviewers could provide clarity on concepts that were not understood by participants.

**Step 4:** Participants in step 2, who displayed the least understanding of the messages under review, participated in three workshops (n = 22) to evaluate the refined text messages. Each workshop had between 5 and 10 participants. The project manager (intervention developer) conducted the workshop with two interviewers as co-facilitators in step 3.

**Step 5:** The experts used the notes derived from step 4 to finalize the messages in the text bank.

## 3. Results

### 3.1. Evaluation of Text Messages for Readability/Understandability and Acceptability in Terms of Content, Language, and Quality of Information

Overall, all text messages were well received and scored high for their understandability and acceptability. Between 87.5% and 100% of the participants selected “agree” to the quality of the information captured in the text messages. Text messages that scored poorer had content and language components: <80% of participants selected “agree” for the content of 32 (48%) and language of 19 (28%) text messages, respectively. The messages that fared the worst in terms of both content (i.e., less than 60% of the participants selecting “agree”) and language (i.e., less than 65% of the participants selecting “agree”) were all related to physical activity/exercise. Of the nutrition-related messages, the message “Eat food from different groups everyday” fared the worst with regards to content (only 52.6% of the participants selected “agree”) although 80.3% of the participants selected “agree” on the language component of the message, see Supplementary SMS paper Graphs.

The qualitative feedback provided context and explained what participants did not understand about certain messages or how they interpreted them. Examples of comments recorded during the telephonic and in-person interviews are illustrated below:

“Explain dark green leafy” [SMS 2—Eat a dark green leafy vegetable and an orange fruit or vegetable daily].

“Groups not clear, thought it was different times of the day” [SMS 4—Eat foods from different groups every day].

“Variety not clear” [SMS 7—An easy way to increase variety and vegetable intake is to eat a fresh salad with your cooked meal].

### 3.2. Data Collation and Refinement of Messages

Using the above feedback, an expert panel consisting of four nutrition experts, including the two who conducted the interviews in step 2, two physical activity specialists, and the project manager, refined the 37 less understood/acceptable/applicable text messages. The panel members were guided by the qualitative interview data as well as their knowledge and experience acquired from working with low-resourced communities. A few examples of refined messages are listed below in Table 1.

### 3.3. Participant Validation of Refined Messages

Following the refining of the text messages, three workshops were held to evaluate refined messages. Overall, the feedback from the workshops was positive. Several of the issues raised by the participants were related to the practicality of the text messages rather than them not being well understood. For example, feedback on refined messages 3–5 listed in Table 2 indicates that the participants viewed these messages as not being practical.

### 3.4. Text Bank Finalized

Following the participant validation of the 37 refined messages, 7 messages were further amended based on participant feedback; examples are provided in Table 3. The final text bank consists of 67 messages, with topics related to fruit and vegetable consumption, as well as variety in the diet (n = 15), limiting fat intake (n = 10), avoiding sugar (n = 11), avoiding salt (n = 5), promoting fiber-rich foods (n = 1), messages promoting physical activity (n = 21), and general check-in messages (n = 4).

## 4. Discussion

This paper describes the process of developing text messages for people at risk of diabetes as part of the SA-DPP lifestyle intervention program. There were 67 text messages designed to encourage healthy behavioral changes in people at risk for diabetes. Increasing evidence suggests that the addition of text messaging to standard healthy lifestyle interventions may improve the outcomes of DPPs as well as other programs related to health behaviors [9,10,11]. The use of text messaging seems to be an ideal platform because text messages are read within minutes of being received and could be helpful as they provide short, timed bursts of information, which are constant reminders of behavior change goals, at a low cost [14,15].

Our processes for the development of our text messages conform with the suggestions by Willoughby and Furberg (2015) [14]. In their review, they recommend the use and modification of existing, empirically tested content and pretesting of messages with the target audience. In this study, first, the initial text messages were developed using the content of an already adapted diabetes prevention curriculum suited to the target audience [21]. These messages were informed by the SAFBDGs, which were developed specifically for and tested, albeit to a limited extent, in the SA population [26,27,28,29]. Second, the target population participated in the pretesting of the text messages.

The processes in this study ensured flexibility and responsiveness to the messages with multiple feedback sessions from the target population. During the pretesting of the text messages, participants assessed the language of the message for understandability, the content for relevance, and the overall quality of the text messages. Text messages with low agreement for adequate language and content were refined for easier understanding and greater acceptability. These refined messages were again reviewed by the target audience. The testing of messages in the SA context is especially important as it has a diverse population with many different cultures, religions, and 11 official languages. Authors have indeed recognized that studies should ensure that text messages are written appropriately for the target population [30]. If the target population does not identify with or enjoy the content of a health behavior change program, they may disengage from the program [15].

The testing and refinement process elicited important information to consider when developing text messages for a specific target population. During the development process, terms or concepts unfamiliar to the study participants were clarified by either giving examples (e.g., examples of different colored fruits and vegetables) or rephrasing terms. Study participants did not understand the phrase “wilted vegetables” in the original message; neither did they understand the revised phrase “stale vegetables”, so the wording was further simplified to read “unused or leftover vegetables”. Keeping the language as simple as possible is, therefore, key to ensuring that the messages are easy to understand. Furthermore, the target population needs to be able to identify with the practice promoted in the message. For example, the suggestion of including a fresh salad with a cooked meal was not common among our target group, so the text message was revised to “Eat a salad with your meal to increase your vegetable intake”.

Participants also struggled with the complete avoidance of sugar and processed foods. Resistance to dietary change could be because of cost and taste preference. Processed foods such as polony can be limited but not avoided as polony is a cheap source of protein. Similarly, Love et.al. found barriers to the implementation of the SAFBDGs to be the cost of foods, taste preferences, and the confusion around concepts and certain terminology used in messages [26,27]. Other barriers are limited access to resources and cultural or family practice constraints [28]. Factors that influence dietary choices such as availability, affordability, and preference should be considered when defining nutrition messages [24]. In assessing acceptability prior to an intervention, the participants’ perspectives on the content and perceived quality of the intervention were considered. When an intervention was deemed acceptable, and participants were part of the process they were more likely to adhere to the intervention recommendations and benefit from it [31].

A strength of this study is that it followed recommendations from the literature which highlighted the importance of formative assessments (step 1), pretesting (step 2), and validation of text messages (step 4) as part of the development process for health behavior change interventions [13,14,15,16]. This study addresses the gap in the literature where documentation of development processes in studies is mostly lacking [14]. The process outlined in this study can serve as a guide for researchers and intervention developers who wish to develop text messages for health interventions.

Limitations of the study include the relatively low number of participants available for interviews in step 2 due to COVID-19 restrictions. Also, this process was restricted to two population groups in Cape Town, Western Cape, which is different from other demographical areas in SA, both culturally and economically. However, to temper the effect of these limitations further efforts to validate the SA-DPP intervention (including text messages), an extension of this program is currently starting in Eastern Cape [32]. We are also adapting the intervention for people living with HIV in another study pertaining to the cardiometabolic effects of weight gain associated with antiretroviral treatment.

## 5. Conclusions

This paper describes the process of developing text messages focused on behavior change for people at risk of diabetes in low-resourced communities as part of the SA-DPP intervention. The final text messages were deemed acceptable and practical by the target population, which would make intervention adherence and retention more likely. The text message library is readily scalable and, if successful, can be adapted for implementation in similar populations.

## Figures and Tables

**Table 1:** Examples of refinement of messages using information from interviews.

Original Message	Comments Received (Survey as Recorded by Interviewers)	Refined Message
Eat vegetables of different colors, e.g., dark green leafy, yellow, orange, and purple vegetables.	Beetroot is not a purple veg. See it as red.	Eat vegetables of different colors, e.g., dark green leafy (spinach), yellow, orange (butternut/carrots), red (tomato), and purple vegetables (beetroot).
Eat different types of food daily to ensure variety in your diet.	“Different types of food” was understood as different vegetables. Diet was understood to mean eating a “certain way”.	Eat foods from different groups every day, i.e., meat, dairy, vegetables, and fruit.
Limit or avoid processed foods such as polony and sausage. Healthier options are eggs, peanut butter, canned tuna, and pilchards.	Processed foods were not commonly understood.	Limit ready-to-eat meat products such as polony, Vienna sausages, and Russian sausages. Healthier options are eggs, peanut butter, canned tuna, and pilchards.
Do not throw away stale vegetables, use them in soups and stews.	54.5% of participants did not understand the term “stale”	Do not throw away wilted vegetables, use them in soups and stews.
Eat a fresh salad with your cooked meal to add variety and increase your vegetable intake.	Do not usually eat salad with cooked food.	Eat a salad with your meal to increase your vegetable intake.
Every hour of TV you watch may shorten your life by 22 min. Why not exercise instead? It can lengthen your life!	Did not understand.	Sitting and watching too much TV is not good for your health. Why not exercise instead?

**Table 2:** Examples of participant feedback on refined messages.

Refined Message	Comments Received from Participants
Do not throw away wilted vegetables, use them in soups and stews.	Wilted not clear to all. “Wilted sounds very up-pity”. In Afrikaans, the group understands/or sees it as “oorryp” [over ripe] [Notes from Workshop 1] Wilted” not clear to most participants. [Notes from Workshop 2] “Wilted” not understood by most participants. [Notes from Workshop 3]
2. Slowly reduce salt in food: herbs and spices can be used to make food tasty.	Spices are seen as chicken spice and barbeque spice, which already contain salt. Herbs are rarely used/only in specific dishes. [Notes from Workshop 1] Herbs were understood correctly. Spices were understood to be “Aromat” or “Braai spice”. Rather say “natural spice” and give examples. [Notes from Workshop 2] Herbs were understood as “plants”. Spices were understood to be Robertson’s “steak & chop spice” or “chicken spice”. [Notes from Workshop 3]
3. Increase your fiber/roughage intake, enjoy oats or Weetbix/whole wheat cereal/bran without sugar for breakfast.	Problem with eating cereal with no sugar. Suggestion that we say less sugar to no sugar as was done with a message with reference to tea and coffee. [Notes from Workshop 1] Not all understood “roughage/fiber” There was much resistance to “without sugar for breakfast”. They cannot have Weetbix without sugar. [Notes from Workshop 2] Most did not understand “roughage/fiber”. They have an Xhosa name for it, perhaps that could be used? Participants indicated that Weetbix does need a bit of sugar. [Notes from Workshop 3]
4. Limit or avoid vetkoek with filling—such as polony.	Replied that they cannot avoid it, only limit it. [Notes from Workshop 2] Replied that they cannot completely avoid it. [Notes from Workshop 3]
5. Avoid sweets and chocolates as they increase your blood sugar too much and too quickly.	Replied that they cannot completely avoid it, but they can limit it. [Notes from Workshop 2] Replied that they cannot completely avoid it. [Notes from Workshop 3]

**Table 3:** Examples of final messages based on participant feedback.

Refined Message	Final Message/s
Do not throw away wilted vegetables, use them in soups and stews.	Do not throw away unused or leftover vegetables, use them in soups and stews.
2. Slowly reduce salt in food: herbs and spices can be used to make food tasty.	Slowly reduce salt when preparing food: use herbs, salt-free spices, and seasonings instead to make food tasty.
3. Avoid salty snacks such as Niknaks and chips (Simba).	Avoid salty snacks such as Niknaks and chips (Simba) to lower your salt intake.
4. Sweets and chocolates increase your blood sugar excessively and should therefore be avoided.	Avoid sweets and chocolates as they increase your blood sugar too much and too quickly.
5. Every new week, every new day brings a new opportunity to start or restart healthy habits.	Every new week brings new opportunities to start or restart healthy habits. Every day brings new opportunities to live healthily.

## Data Availability

Not Applicable.

## Supplementary Materials

The following supporting information can be downloaded at: https://www.mdpi.com/article/10.3390/nu15214692/s1, SMS Paper Graphs.
