# Supplementary material for: The Development of Text Messages to Support People at Risk of Diabetes in Low-Resourced Communities: The South African Diabetes Prevention Programme
Source: Nutrients. 2023 Nov 5;15(21):4692. doi: 10.3390/nu15214692 (PMC10647382; doi:10.3390/nu15214692)
Supplement: Supplementary file 1 [file nutrients-15-04692-s001.zip › nutrients-2670434-supplementary.pdf]

GROUP 1

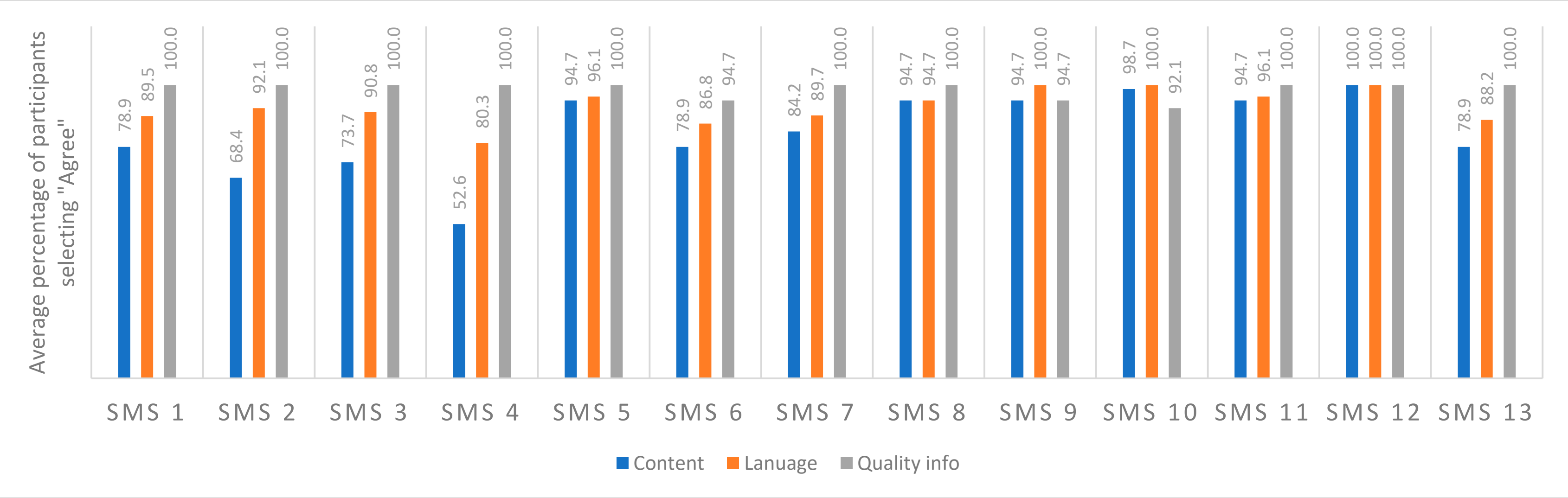

SMS 1 - Eat vegetables of different colours e.g. dark green leafy, yellow, orange and purple vegetables.

SMS 2 - Eat a dark green leafy vegetable and an orange fruit or vegetable daily.

SMS 3 - Eat a variety of foods; include different types of foods daily.

SMS 4 - Eat foods from different groups every day.

SMS 5 - Fruit and vegetables come in rainbow/many different colours, eat a variety of seasonal fruit and vegetables.

SMS 6 - Eat a fresh salad with your cooked meal to add variety and increase your vegetable intake.

SMS 7 - An easy way to increase variety and vegetable intake is to eat a fresh salad with your cooked meal.

SMS 8 - Include vegetables to meals as an easy way to add variety.

SMS 9 - For a cooked meal, ideally half of the plate should be vegetables.

SMS 10 - Use beans or lentils instead of meat in meals as this is a more affordable source of protein.

SMS 11 - Eat 3 vegetables and 2 fruits daily to reduce your risk of diseases such as diabetes. In general, South Africans eat less than half of this recommended amount.

SMS 12 - Make sure you eat vegetables and fresh fruit every day.

SMS 13 - Have you thought of your food/exercise/healthy lifestyle goals for the week ahead?

GROUP 2

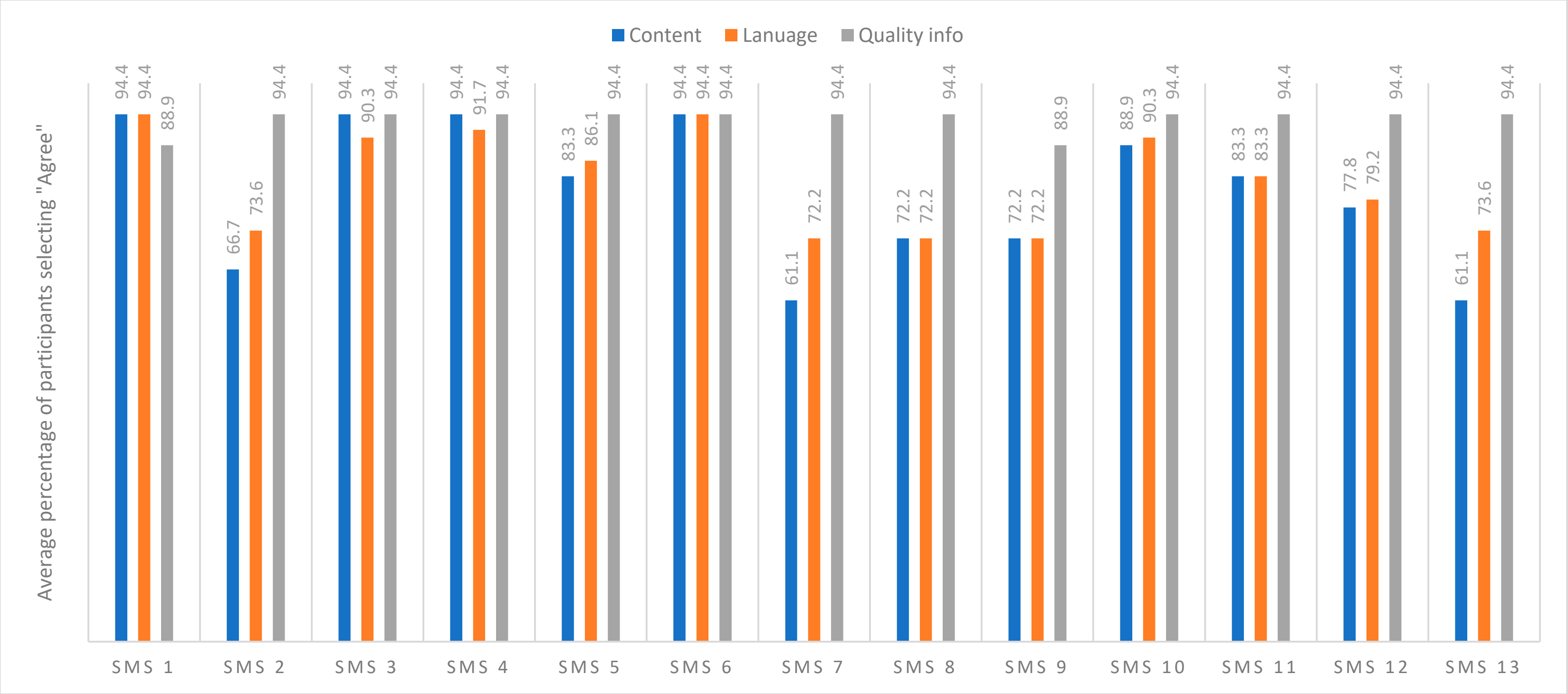

SMS 1 - Buy vegetables and fruit from street vendors as they are usually cheaper than the supermarket.
SMS 2 - Do not throw away stale vegetables, use them in soups and stews.
SMS 3 - Frozen mixed vegetables are an excellent way to eat a variety of vegetables of different colours.
SMS 4 - When you have a dessert with your meal, rather eat a fresh fruit or fruit salad.
SMS 5 - Avoid salty snacks such as Niknaks and chips.
SMS 6 - Avoid deep fried foods such as hot chips, vetkoek, koeksisters and samosas.
SMS 7 - Limit or avoid vetkoek with filling – such as polony or processed cheese.
SMS 8 - Limit or avoid processed foods such as polony and sausage. Healthier options are eggs, peanut butter, canned tuna and pilchards.
SMS 9 - Use soft margarine rather than hard margarine as this is healthier but use it sparingly.
SMS 10 - To reduce your fat intake, remove the skin and visible fat from chicken, before cooking.
SMS 11 - To reduce your fat intake, remove the visible fat from meat before cooking.
SMS 12 - In meat dishes replace some of the meat with beans or lentils to reduce your fat intake.
SMS 13 - Have you thought of your food/exercise/healthy lifestyle goals for the week ahead?

GROUP 3

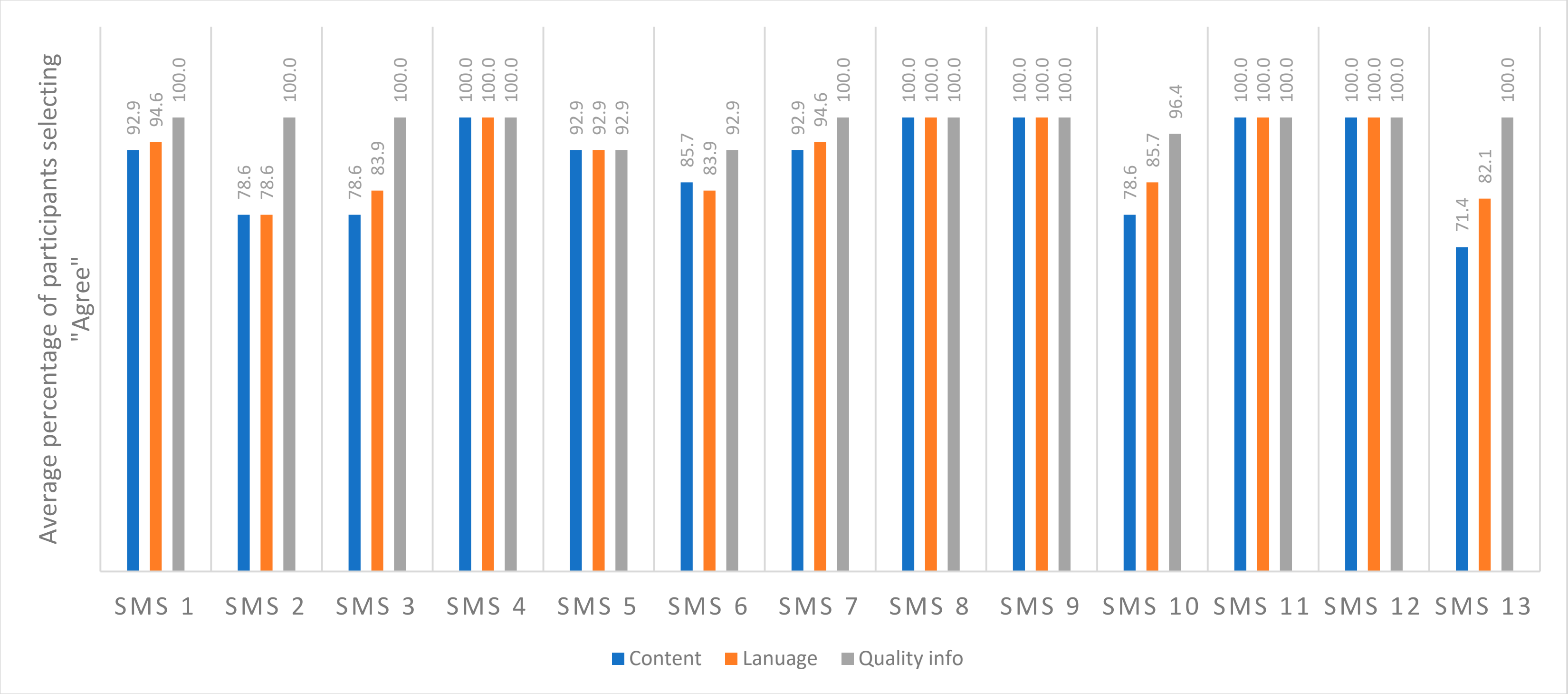

SMS 1 - Milk is a good source of calcium which is important for strong bones – low fat milk is a good option.
SMS 2 - Fizzy drinks and cordials contain a lot of sugar and should therefore be avoided.
SMS 3 - Limit the intake of fruit juice as they are high in natural sugars. One glass of unsweetened orange juice has the same amount of sugar as three oranges.
SMS 4 - Drink water rather than cold drinks and juice.
SMS 5 - Water is essential for a healthy body, aim for 8 glasses per day.
SMS 6 - Gradually reduce your sugar added to tea and coffee to preferably zero.
SMS 7 - Brown sugar and honey is very similar to white sugar and should therefore be avoided.
SMS 8 - Do not add sugar to stews and curries.
SMS 9 - Do not add sugar, oil or margarine to vegetables.
SMS 10 - Sweets and chocolates increase your blood sugar excessively and should therefore be avoided.
SMS 11 - Cake, biscuits and doughnuts are high in sugar and should be avoided. At special occasions, limit yourself to one small serving.
SMS 12 - At special occasions serve healthier options such as fruit on a stick instead of cake.
SMS 13 - Are you ready for your food/exercise/healthy lifestyle goals this week?

GROUP 4

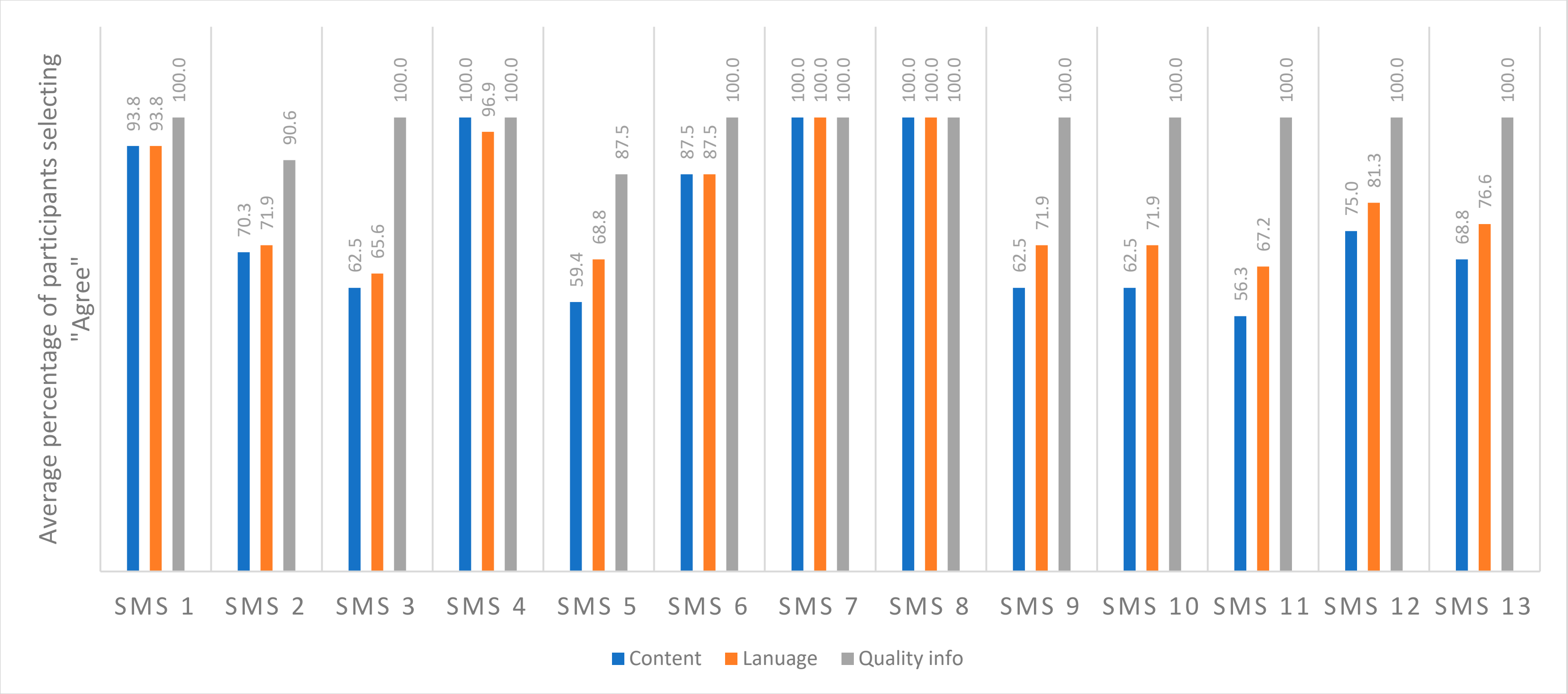

SMS 1 - Reduce your salt intake by not adding extra salt or aromat to your food at the table.
SMS 2 - Be aware that powdered soups and most canned foods contain a lot of salt, use these sparingly.
SMS 3 - Gradually/slowly reduce salt in food by experimenting with herbs and spices to get the most enjoyable taste for you and your family.
SMS 4 - Do not add salt to vegetables when cooking, vegetables has its own natural flavours.
SMS 5 - Increase your fibre intake, enjoy oats or Weetbix without sugar for breakfast.
SMS 6 - Daily exercise helps control our weight, feel better, have energy and fight diseases.
SMS 7 - Just 10 minutes of exercise (walks, aerobics, dance) a couple of times a day makes a big difference to one's health.
SMS 8 - Exercise regularly to reduce stress.
SMS 9 - Stress is a daily occurrence. Remember to exercise regularly to reduce stress.
SMS 10 - Stay active. Even a short, brisk walk every day can make a difference.
SMS 11 - Limit sedentary behavior (sitting for too long). Remember to get up and stretch, stand from time to time, and go for a short walk, when sitting for too long.
SMS 12 - Increase your physical activity. Find activities that you enjoy i.e. walking with a partner/group, dancing, yoga or family games.
SMS 13 - Are you ready for your food/exercise/healthy lifestyle goals this week?

GROUP 5

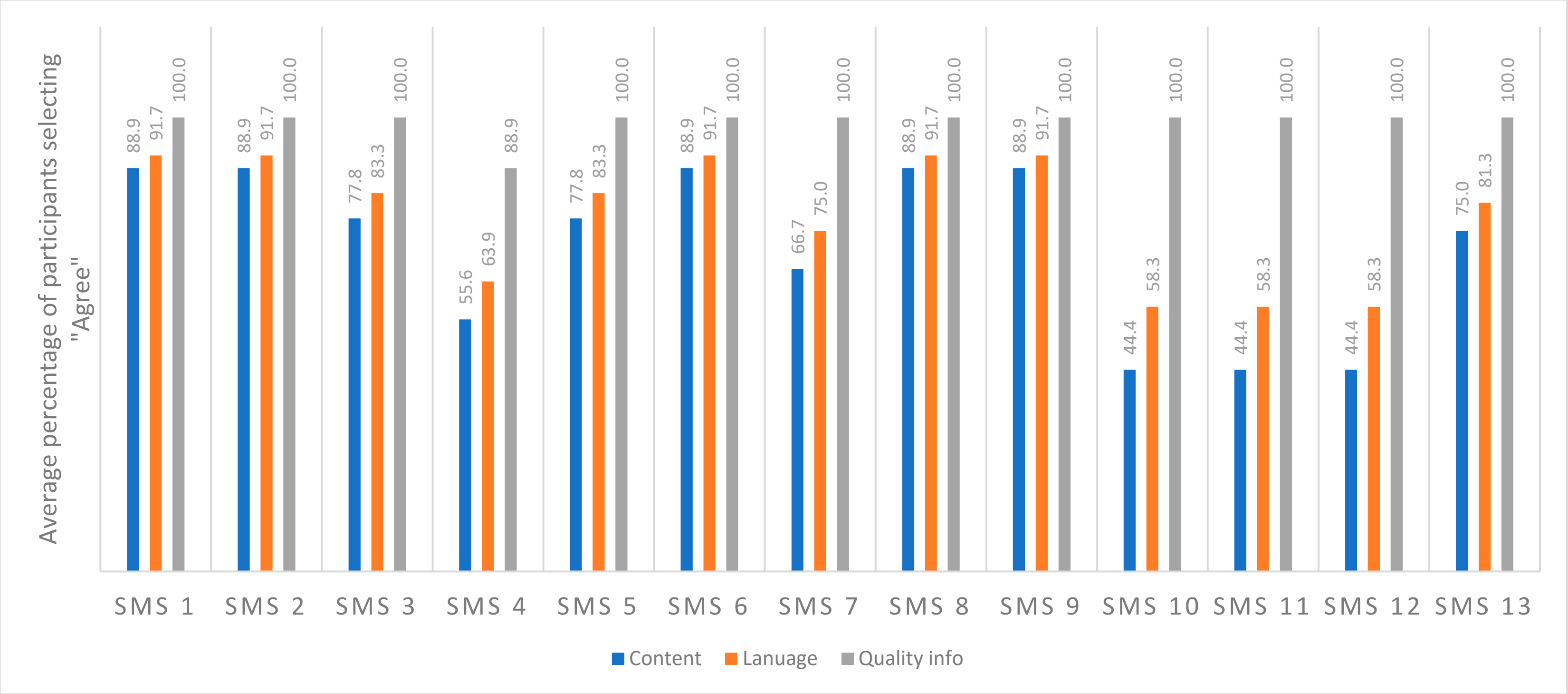

SMS 1 - Exercise promotes good health. Aim for at least 30 minutes of physical activity most days.
SMS 2 - Exercise is good for people of all ages. Get the whole family involved.
SMS 3 - Exercising in a group keeps you motivated. Include your family and friends.
SMS 4 - Every hour of TV you watch may shorten your life by 22 minutes. Why not exercise instead? It can lengthen your life!
SMS 5 - When bored instead of snacking go for a walk.
SMS 6 - Sit less. Move more. Stay active.
SMS 7 - Get the most out of your day by being physically active every day.
SMS 8 - Exercise is a lot more fun with a friend, invite a friend along with you.
SMS 9 - Exercise increases blood flow which leads to a healthier heart.
SMS 10 - Daily exercise improves mental/overall wellness.
SMS 11 - Daily exercise improves balance and coordination. Keep moving.
SMS 12 - Keep active. Remember exercise lowers your blood pressure and regulates your insulin (sugar levels).
SMS 13 - A healthy lifestyle is a balanced lifestyle, being active helps us achieve this.
